# Thoracic day surgery versus thoracic inpatient surgery for treatment of patients with lung cancer: a systematic review and meta-analysis

**DOI:** 10.1186/s13019-023-02462-4

**Published:** 2023-11-25

**Authors:** Cheng Shen, Guowei Che

**Affiliations:** Department of Thoracic Surgery, West-China Hospital, Sichuan University, Chengdu, 610041 China

**Keywords:** Day surgery, Enhanced recovery after surgery, Lung cancer, Video assisted thoracic surgery, Complication

## Abstract

**Background:**

The successful implementation of the Enhanced recovery after surgery (ERAS) concept in thoracic surgery has made it possible to complete the surgery in the day surgery unit. However, it is still unclear whether day surgery for lung cancer patients can achieve the same or even better results.

**Methods:**

A systematic literature search was completed in common databases for studies published before May 2022 and the data analyzed using the Review Manager 5.3 software.

**Results:**

We ultimately included 7 retrospective articles that met our criteria for the study. The results of age, smoking status, comorbidity and pulmonary function tests in day surgery group were better than in inpatient surgery group. Minimally invasive surgical method with segmentectomy was more used in day surgery group than in normal surgery group. The 30-day mortality was also lower in Day surgery group and it displayed that early discharged patients had fewer overall complications than the inpatient surgery group.

**Conclusions:**

We demonstrate that younger patients, patients receiving segmental resections by VATS, and those with better pulmonary function tests or without comorbidity can be discharged early with low rates of complications and 30-day mortality, especial with ERAS program.

## Introduction

Lung cancer is currently the most common disease and one of the leading causes of death in worldwide. In addition, with the popularization and application of low-dose CT (LDCT), more and more young patients are found with small pulmonary nodules in CT [1]. In today’s cost-conscious healthcare environment, it is critical to strike a balance between delivering high-quality healthcare How to provide high-quality medical protection, while also allowing patients to be discharged safely and shortening the length of stay (LOS), is a problem we have been exploring [2]. Enhanced recovery after surgery (ERAS) has been developed furtherly in thoracic surgery [3]. The incidence of perioperative complications and LOS of patients with lung cancer have been reduced obviously by ERAS program.

Day surgery refers to the discharge of a patient undergoing surgical treatment and discharged from the hospital on the first day after surgery through a preoperatively planned and accurately assessed surgical procedure [4, 5]. Although there are several reports on day surgery in patients with lung cancer, they are all retrospective single-center clinical studies [6–8]. Consequently, it is still unclear whether day surgery for lung cancer patients can achieve the same or even better results. We firstly performed this meta-analysis to explore and compare the outcomes of thoracic day surgery versus inpatient surgery.

## Methods

### Search strategy

A systematic literature search was completed in PubMed, Cochrane Library, EMBASE, CNKI, Medline, and Web of Science for studies published before May 2022. The key words used are as follows: (Enhanced recovery after surgery OR ERAS OR Fast track) AND (lung cancer OR lung carcinoma OR lung neoplasm OR lung malignancy) AND (day case OR day-case OR day surgery or day-surgery OR day-stay OR outpatient OR out-patient).

The inclusion criteria: (1) Studies comparing thoracic day surgery with inpatient surgery in patients with lung cancer; (2) Full-text articles including at least one of the following outcomes: operation time, average hospital cost, 30-day readmission, 30-day mortality, postoperative complications.

The exclusion criteria: (1) Review articles, case reports, letters to the editor, comments and meeting reports. (2) Non-human subject studies. (3) Studies without necessary data for statistical analysis. (4) The patients did not undergo day surgery. (5) Non-English Article.

### Quality assessment

The guideline of Newcastle–Ottawa Scale (NOS) was used for evaluating this research. The assessment tool including the star system was used in this research. Specific evaluation system is that 8–9 stars are high quality; 6–7 stars are reasonable quality, and 6 stars less are bad.

### Data collection

Two investigators separately collected relevant data from each included study. Any ambiguities or inconsistencies that arise during the data collection process are addressed through brainstorming. Excel in Tables 1, 2, 3, 4 and 5 is used to collect the basic information.

**Table 1:** Characteristics of the included studies

References	Country	Design	Study period	Group	Cases	Sex (M/F)	Age	Smoking	Comorbidity			
									Hypertension	Diabetes	Coronary heart disease	COPD
Dong et al. [9]	China	R	2019	DG	20	4/16	36.3 ± 11.7	2	1	0	0	1
				NG	28	7/21	43.8 ± 13.2	2	2	1	0	0
Patel et al. [4]	USA	R	2011–2019	DG	854	348/506	66.9 ± 2.5	223	484	147	0	129
				NG	16,064	6668/9394	67.9 ± 3.5	5396	9521	2610	75	3364
Drawbert et al. [2]	USA	R	2010–2015	DG	3819	1626/2193	68.5 ± 6.7	–	–	–	–	–
				NG	3819	1613/2206	68.7 ± 4.8	–	–	–	–	–
Linden et al. [7]	USA	R	2012–2017	DG	1821	773/1048	66 ± 3.5	–	1047	323	290	544
				NG	44,504	19,180/25323	68 ± 5.9	–	27,226	8318	9034	15,816
Dong et al. [6]	China	R	2020–2021	DG	136	25/111	43.30 ± 9.26	10	2	2	–	0
				NG	217	40/177	42.76 ± 10.66	18	5	3	–	0
Towe et al. [8]	USA	R	2007–2017	DG	448	204/244	62.3 ± 7.65	–	206	56	57	–
				NG	613	273/340	64.87 ± 8.15	–	326	109	110	–
Geraci et al. [10]	USA	R	2018–2020	DG	134	48/86	68.5 ± 6.7	72	60	18	14	7
				NG	119	50/69	70.2 ± 7.8	50	75	33	18	9

*R* retrospective study; *DG* day surgery group; *NG* normal surgery group; *COPD* chronic obstructive pulmonary disease; *M* male; *F* female; –, not available

**Table 2:** Characteristics of the included studies

References	Pulmonary function tests		Surgical methods		Operation approach		Tumor location					
	FEV1% predicted	DLCO % predicted	Open	Minimally invasive	Lobectomy	Segmentectomy	RUL	RML	RLL	LUL	LLL	Other
Dong et al. [9]	–	–	0	20	10	10	–	–	–	–	–	–
	–	–	0	28	17	11	–	–	–	–	–	–
Patel et al. [4]	–	–	84	770	–	–	–	–	–	–	–	–
	–	–	72	10,617	–	–	–	–	–	–	–	–
Drawbert et al. [2]	–	–	1811	1400	–	–	1211	348	666	925	583	86
	–	–	2068	1646	–	–	1206	315	677	906	617	98
Linden et al. [7]	88.36 ± 10.45	78.65 ± 20.16	152	1669	1494	327	–	–	–	–	–	–
	84.25 ± 12.17	74.14 ± 18.35	14,275	30,229	39,943	4561	–	–	–	–	–	–
Dong et al. [6]	–	–	–	–	51	84	43	14	15	48	16	
	–	–	–	–	92	124	75	20	31	63	28	
Towe et al. [8]	85.31 ± 8.98	83.90 ± 15.21	2	446	–	–	–	–	–	–	–	–
	75.62 ± 7.42	73.21 ± 13.96	33	580	–	–	–	–	–	–	–	–
Geraci et al. [10]	89.12 ± 20.66	82.45 ± 13.12	–	–	53	79	36	12	23	46	15	–
	85.43 ± 16.35	79.89 ± 11.28	–	–	71	37	43	8	25	23	16	–

FEV1% predicted, percent of forced expiratory volume in 1 s predicted; DLCO% predicted, percent of diffusing capacity of the lung for carbon monoxide predicted

*RUL* right upper lobe; *RML* right middle lobe; *RLL* right lower lobe; *LUL* left upper lobe; *LLL* left lower lobe

**Table 3:** Characteristics of the included studies

References	Histology			TNM stage				NOS
	Adenocarcinoma	Squamous carinoma	Other	I	II	III	IV	
Dong et al. [9]	20	0	0	20	0	0	0	8
	27	1	0	27	1	0	0	
Patel et al. [4]	–	–	–	–	–	–	–	7
	–	–	–	–	–	–	–	
Drawbert et al. [2]	2489	764	566	–	–	–	–	8
	2476	760	583	–	–	–	–	
Linden et al. [7]	–	–	–	–	–	–	–	7
	–	–	–	–	–	–	–	
Dong et al. [6]	135	1	–	134	2	–	–	8
	215	2	–	211	6	–	–	
Towe et al. [8]	–	–	–	–	–	–	–	7
	–	–	–	–	–	–	–	
Geraci et al. [10]	85	9	29	76	7	6	–	8
	70	12	20	69	8	11	–	

*NOS* Newcastle–Ottawa scale

**Table 4:** Results of the analysis of two groups

References	Operation time (min)	Average hospital cost ($)	30d Readmission	30d Mortality	Postoperative compliactions								
					Total	Pneumothorax	Hydrothorax	Homorrhage	Arrhythmia	Lung infection	Chylothorax	Persist air leak	Hoarseness
Dong et al. [9]	–	6005.43 ± 534.25	0	0	1	–	–	–	0	–	–	–	1
	–	7500.55 ± 1156.69	0	0	1	–	–	–	1	–	–	–	0
Patel et al. [4]	142.5 ± 10.4	–	51	5	2	2	–	–	–	–	–	–	–
	168.7 ± 17.7	–	1128	47	1	1	–	–	–	–	–	–	–
Drawbert et al. [2]		–	197	90	–	–	–	–	–	–	–	–	–
		–	137	40	–	–	–	–	–	–	–	–	–
Linden et al. [7]	193.0 ± 40.7	–	114	5	92	–	5	5	32	11	1	36	2
	240.0 ± 55.8	–	3485	169	10,537	–	490	1105	4292	805	124	3592	129
Dong et al. [6]	68.81 ± 21.33	6,411.47 ± 657.76	1	0	16	2	1	3	0	1	1	6	2
	98.15 ± 11.34	7,522.41 ± 1,471.84	3	0	25	4	2	4	0	0	2	10	3
Towe et al. [8]	64.80 ± 41.22	–	0	0	–	–	–	–	–	–	–	–	–
	87.25 ± 35.67	–	0	0	–	–	–	–	–	–	–	–	–
Geraci et al. [10]	84.15 ± 15.35	–	1	0	13	–	3	–	3	–	–	7	–
	101.34 ± 21.56	–	4	0	40	–	6	–	10	–	–	24	–

**Table 5:** Summary of all the researches

References	Summary content
Dong et al. [9]	20 patients were included in day surgery (DS) and 28 patients were applied inpatient surgery (IS). The average hospital day in DSgroup was significantly shorter than in IS group. The average hospital cost in DS group was significantly lower than in IS group.There was no significant difference in the incidence of postoperative complications between two groups
Patel et al. [4]	Only 854 (3.8%) of 22,585 patients that met inclusion criteria were discharged with day surgery. A minimally invasive approach wasthe strongest predictor of early discharge. Readmission rates were not significantly different for two groups.
Drawbert et al. [2]	3879 (7.3%) patients were discharged on day 1, whereas 48951 (92.7%) were discharged after day 1. Factors associated with daysurgery included male sex, higher socioeconomic status, right middle lobectomy, minimally invasive surgery and high-volumecentres.
Linden et al. [7]	1821 patients (3.9%) were discharged on day 1. In multivariable analysis, factors associated with day 1 discharge included age, bodymass index greater than 25, forced expiration value at 1 second, middle or upper lobectomy, minimally invasive technique, andprocedure time. Outpatient 30-day mortality was similar in two groups. Patients discharged on day 1 were not at increased risk ofreadmission.
Dong et al. [6]	136 individuals in DS and 217 individuals in IS. With respect to the postoperative complications (PPCs), no difference between thetwo groups was found. In the DS, a shorter length of stay after surgery and reduced drainage time were found, while the drainagevolume per hour (mL/h) was not notably divergent between the relevant groups. No difference was observed in the cost ofequipment and materials between the two groups. However, the average hospital cost and drug cost of the DS were significantlylower than those of the IS.
Towe et al. [8]	DS after lung resection is multifactorial but is safe among selected patients. Age, lung function, procedure duration, and surgeon allinfluence DS. Complications after DS were rare. Education or enhanced recovery protocols may help overcome this barrier.Standardized pathways would likely help identify low-risk patients for expeditious discharge.
Geraci et al. [10]	134 patients (53%) discharged by day 1. On multivariate analysis, never smokers and segmentectomy were associated with DS.Conversely, decreased baseline performance status and perioperative complications were associated with DS. There were 4readmissions (1.6%), of which one (0.4%) was after day 1 discharge. Patient satisfaction remained high throughout the study period.

### Statistical analysis

Review Manager 5.3 software were used for statistical analyses. The dichotomous variables were assessed by using odds ratios (OR) with a 95% confidence interval (CI) and the continuous variables using weighted mean difference (WMD) with a 95% CI. The I^2^ statistics were used to evaluate the heterogeneity. The potential publication bias was evaluated by visually inspecting the funnel plots. *P* < 0.05 was regarded as statistically significant.

## Results

### The selection of included studies

**Fig. 1:**
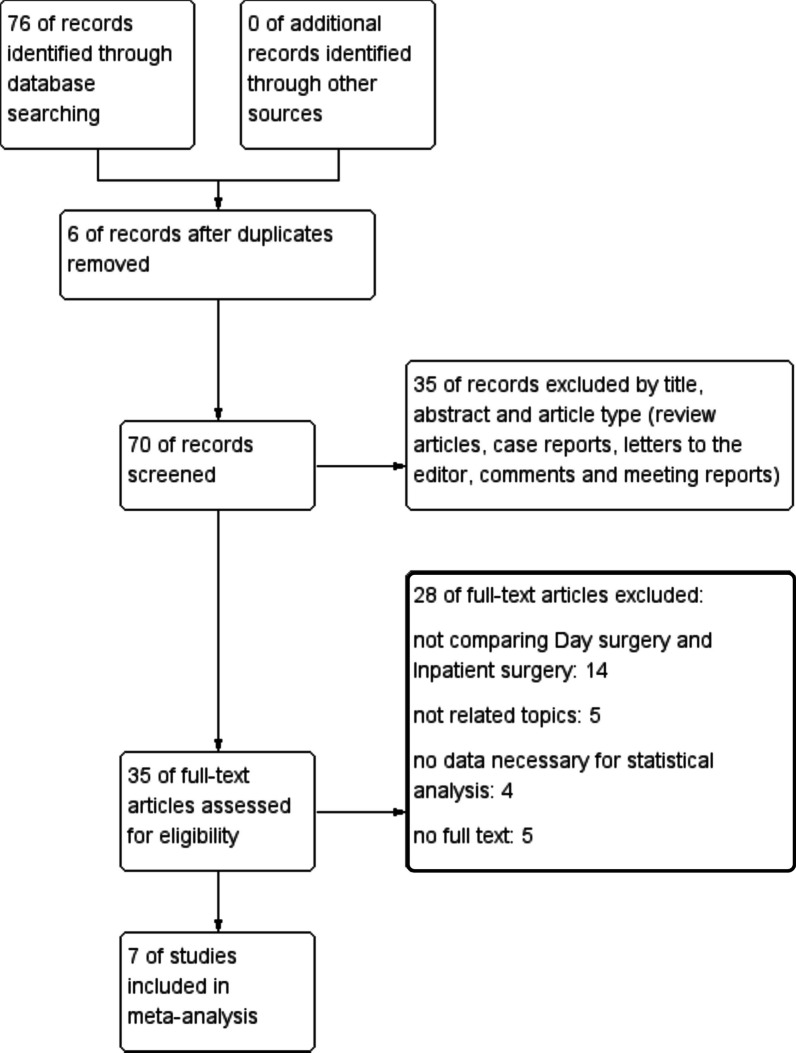
Flow chart of literature search strategies

Databases were searched and total number of studies is 76 before May 2022. After removing 6 duplicate articles, we carefully read the remaining 70 articles. Then, 35 articles were excluded due to article type that did not meet our inclusion criteria. Subsequently, after a detailed reading of the remaining 35 papers, combined with our inclusion and exclusion criteria, 20 papers were finally excluded. In our meta-analysis, we finally included 7 retrospective articles that met our criteria for the study rigorously (Fig. 1).

### The characteristics of included studies

A prospective study was completed by Dong et al. [9]. The results were primarily analyzed for length of hospital stay, hospitalization costs and complications. Patel et al. [4] analyzed the outcomes of patients who were discharged on postoperative day 1 (POD1) with normal approach group. Drawbert et al. [2] summarized patients with stage I non-small cell lung cancer (NSCLC) from 2010 to 2015 and research objects including two groups. In Linden et al. [7] study, they found that carefully selected patients before the surgery may not increase risk of readmission or death. In Dong et al. [6] research, 353 patients were included with 136 persons in the day surgery group and 217 people in the inpatient surgery group. Towe et al. [8] reported that POD 1 discharge patients after lung resection is safe. Geraci et al. [10] evaluated safety for patients discharged by POD1 after different range resection of lobe.

### The age between two groups

**Fig. 2:**
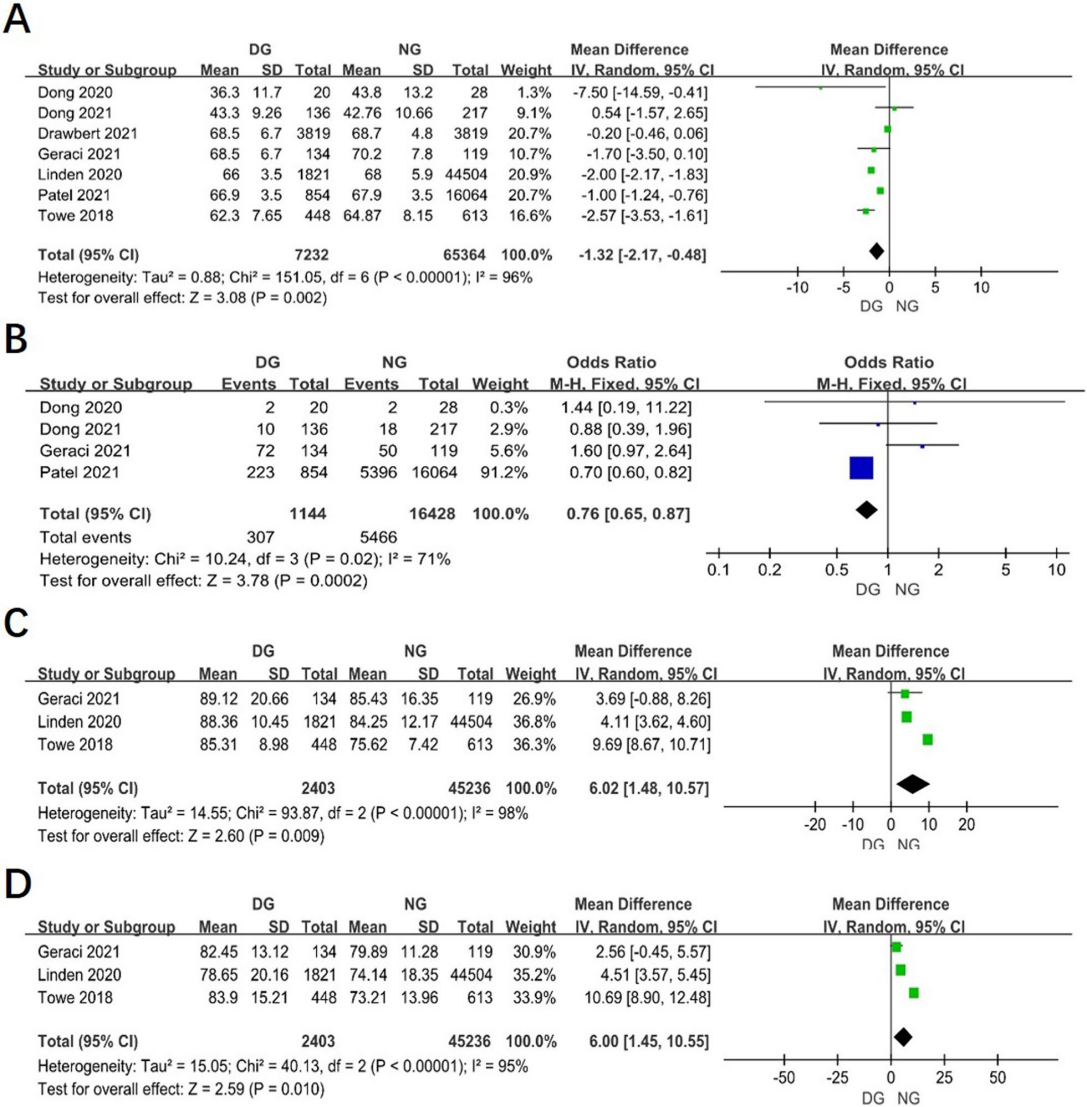
Forest plot of the meta-analysis. **A** Age. **B** smoking status of patient. **C** FEV1% predicted. **D** DLCO% predicted

Age of patients was reported in all studies. The combined data revealed that the age in Day surgery group (DG) was younger than in inpatient surgery group, or named Normal surgery group (NG) (WMD = − 1.32, 95% CI − 2.17 to − 0.48, *P* = 0.002, I^2^ = 96%). (Fig. 2A).

### The smoking status of patient between two groups

Briefly, four studies reported smoking status of patients between two groups. It revealed that the patients with smoking status in day surgery group were less than in normal group (OR 0.76, 95% CI 0.65–0.87, *P* = 0.0002, I^2^ = 71%). (Fig. 2B).

### The pulmonary function tests between two groups

The results of 3 studies revealed that the pulmonary function tests in DG were better than in NG, especially in percent of forced expiratory volume in 1 s predicted (FEV1% predicted) (WMD = 6.02, 95% CI 1.48–10.57, *P* = 0.009, I^2^ = 98%) and percent of diffusing capacity of the lung for carbon monoxide predicted (DLCO% predicted) (WMD = 6.00, 95% CI 1.45–10.55, *P* = 0.009, I^2^ = 95%). (Fig. 2C, D).

### The comorbidity before the surgery between two groups

**Fig. 3:**
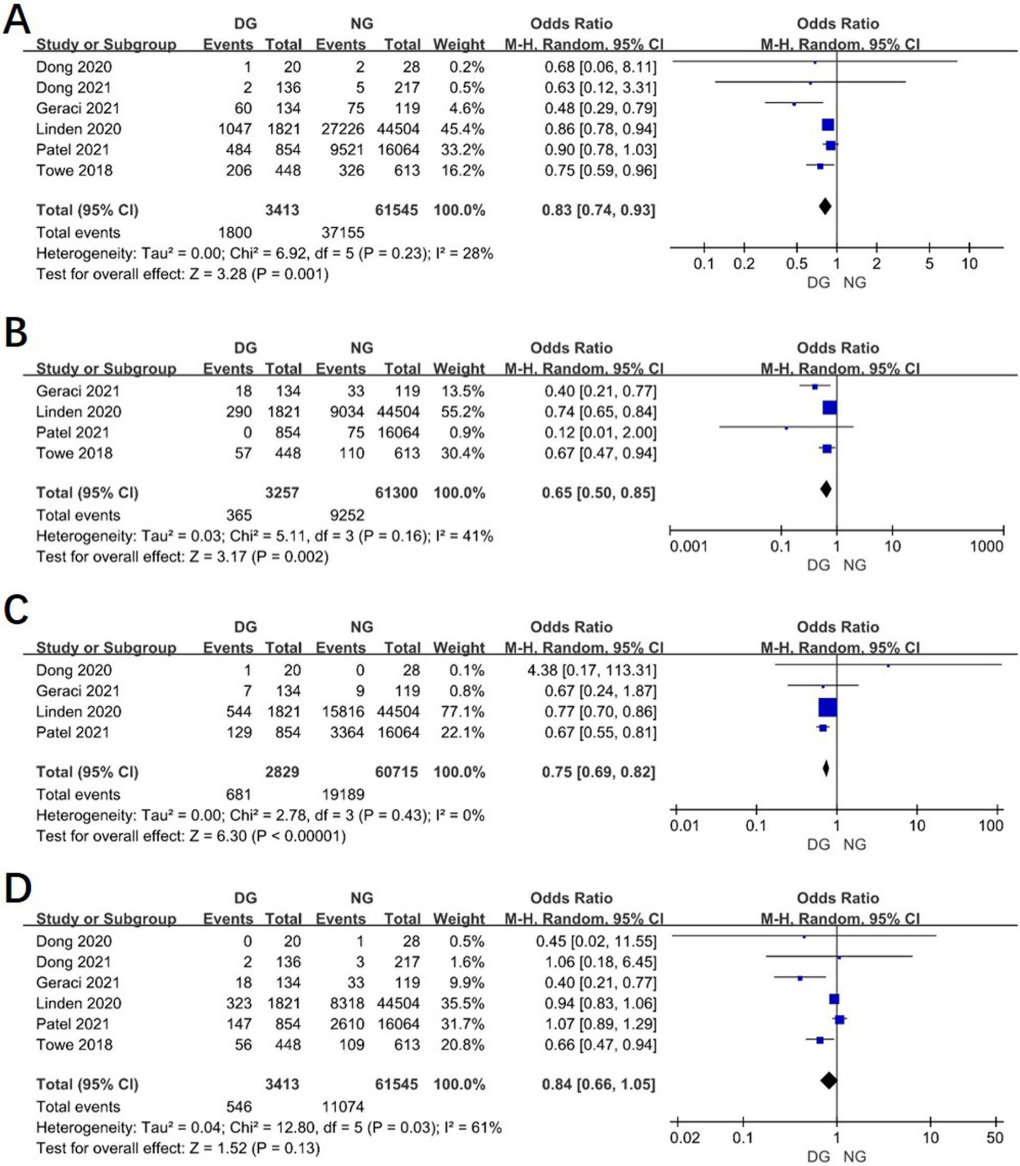
Forest plot of the meta-analysis. **A** Hypertension. **B** Coronary heart disease. **C** COPD. **D** Diabetes

The data regarding the preoperative comorbidity were reported in most of studies. The result showed that patients in day surgery were less likely to have hypertension, coronary heart disease, and chronic obstructive pulmonary disease (COPD) than in normal group. (Fig. 3A, B, C) But there is no difference in patients with diabetes before the surgery in two groups (OR 0.84, 95% CI 0.66–1.05, *P* = 0.13, I^2^ = 61%). (Fig. 3D).

### The surgical methods between two groups

**Fig. 4:**
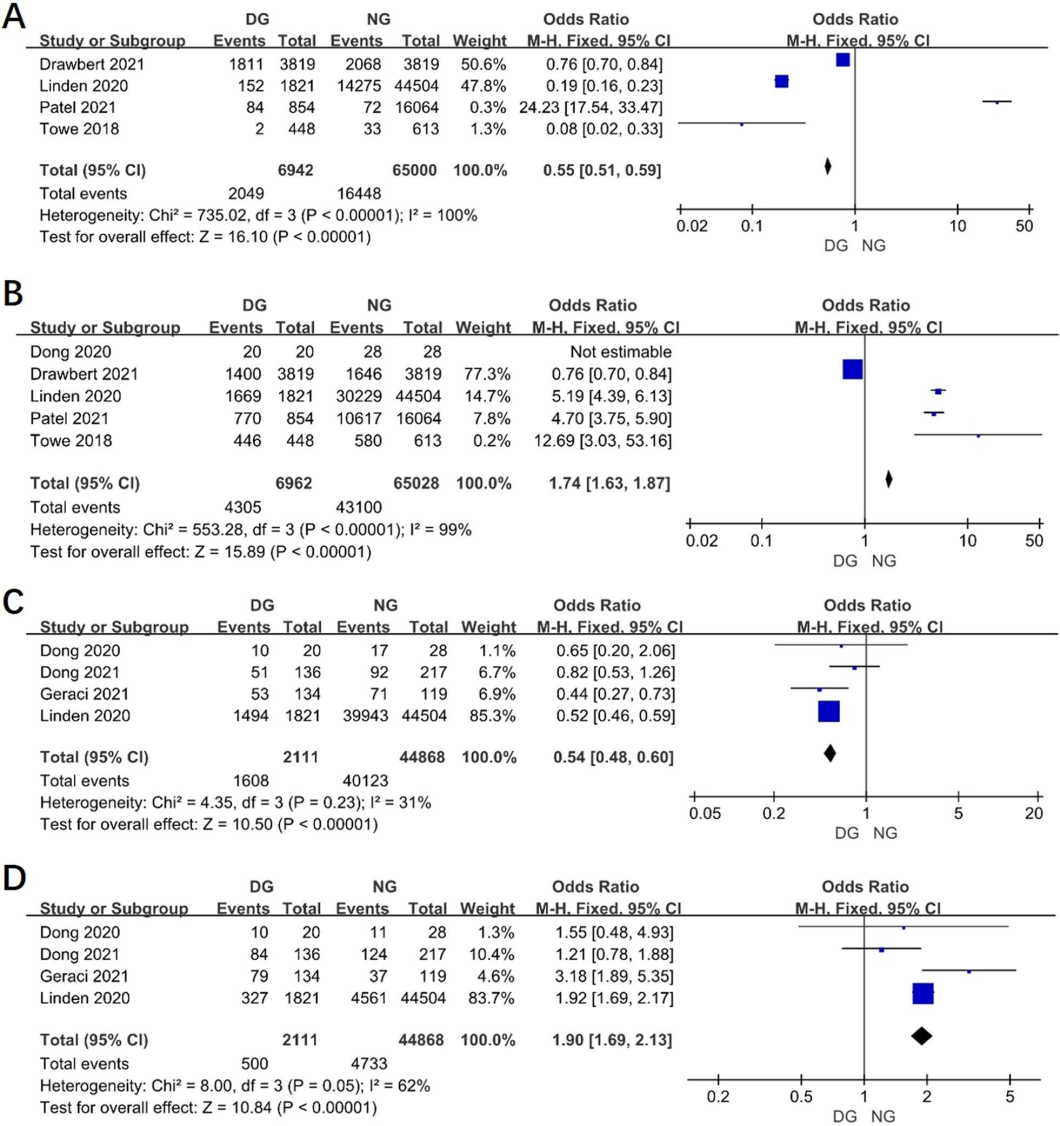
Funnel plot of the meta-analysis. **A** Surgical method by open access. **B** Surgical method by VATS. **C** Lobectomy. **D** Segmentectomy

4 researches reported the surgical method by open access in two groups and the result revealed that open access in DG was less than in NG (OR 0.55, 95% CI 0.51–0.59, *P* < 0.00001, I^2^ = 100%) (Fig. 4A). However, it showed minimally invasive surgical method was more popular in DG than in NG (OR 1.74, 95% CI 1.63–1.87, *P* < 0.00001, I^2^ = 99%) (Fig. 4B).

### The resection range of lobe between two groups

Lobectomy or segmentectomy was informed in 4 studies. The pooled data revealed that the lobectomy in DG was less than in NG (Fig. 4C), but segmentectomy in DG was more than in NG (OR 1.90, 95% CI 1.69–2.13, *P* < 0.00001, I^2^ = 62%) (Fig. 4D).

### The operation time between two groups

**Fig. 5:**
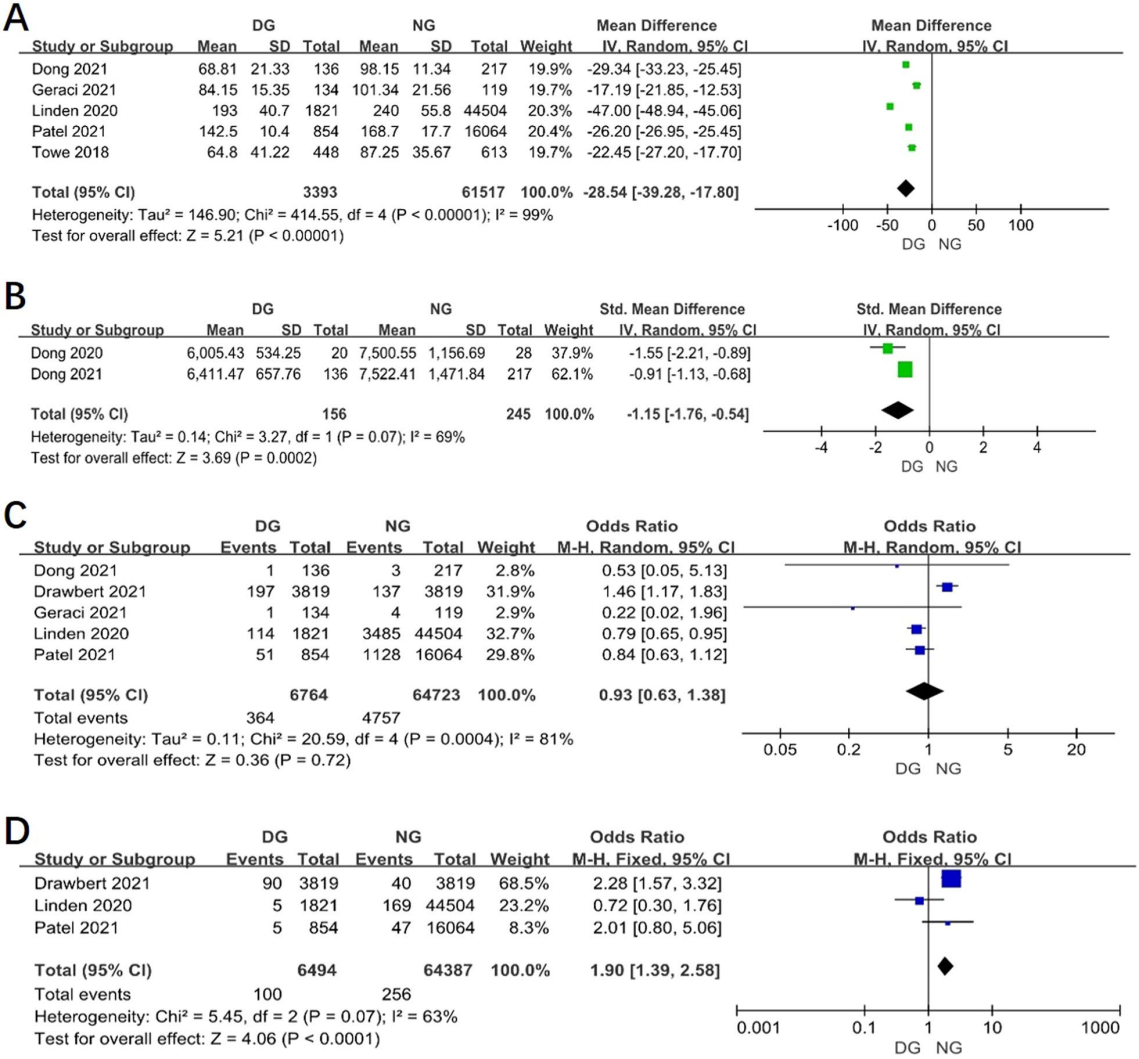
Funnel plot of the meta-analysis. **A** Operative time. **B** Average hospital cost. **C** 30-day readmission. **D** 30-day mortality

Results of 5 studies showed that the operative time was shorter in day surgery group than in normal group (WMD = − 28.54, 95% CI − 39.28 to − 17.80, *P* < 0.00001, I^2^ = 99%). (Fig. 5A).

### The average hospital cost between two groups

According the result of 2 studies, the average hospital cost in DG was lower than in normal group (WMD = − 1.15, 95% CI − 1.76 to − 0.54, *P* = 0.0002, I^2^ = 69%). (Fig. 5B).

### The 30-day readmission between two groups

Results of 5 studies showed that there was no difference between two groups in 30-day readmission (OR 0.93, 95% CI 0.63–1.38, *P* = 0.72, I^2^ = 81%). (Fig. 5C).

### The 30-day mortality between two groups

The 30-day mortality was lower in DG than in NG according to the result of 3 studies (OR 1.90, 95% CI 1.39–2.58, *P* < 0.0001, I^2^ = 63%). (Fig. 5D).

### The postoperative complications between two groups

**Fig. 6:**
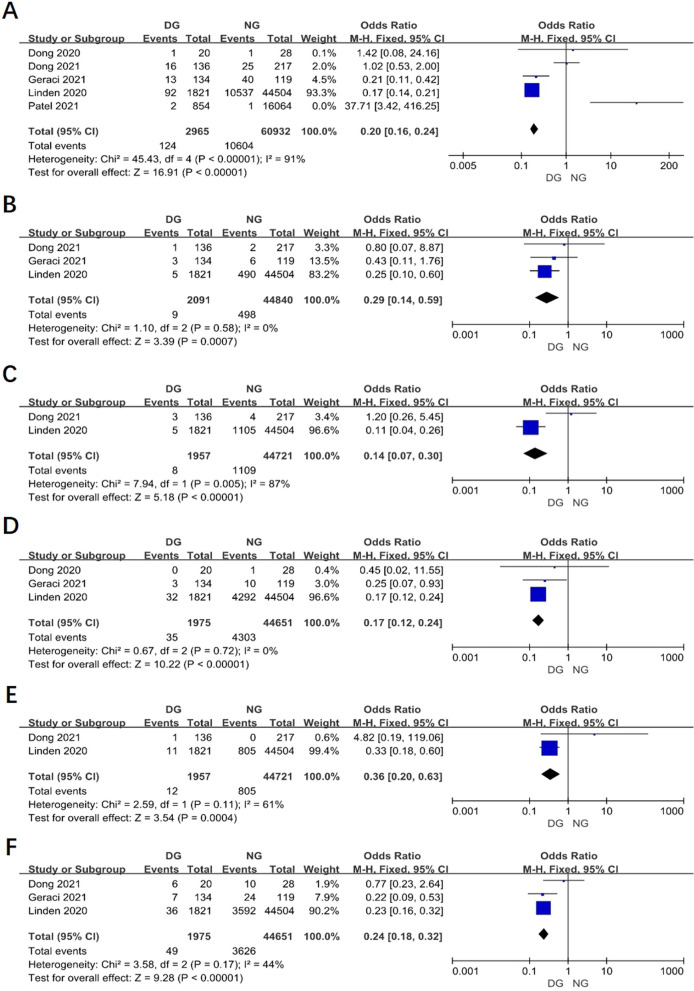
Funnel plot of the meta-analysis. **A** Postoperative complications. **B** Hydrothorax. **C** Hemorrhage. **D** Arrhythmia. **E** Lung infection. **F** Persist air leak

**Fig. 7:**
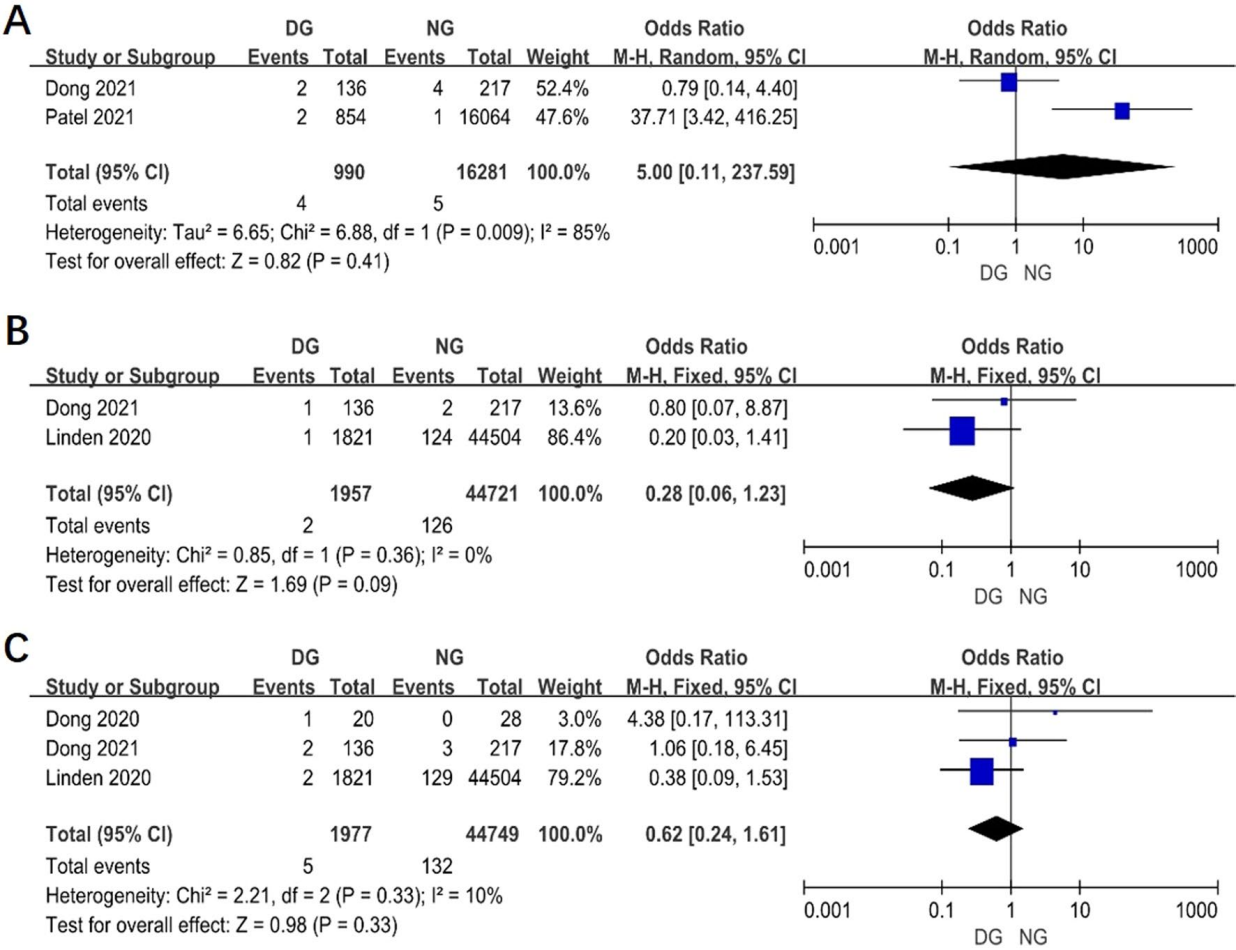
Funnel plot of the meta-analysis. **A** Pneumothorax. **B** Chylothorax. **C** Hoarseness

Postoperative complications of surgery patients was reported in 5 studies. The results showed that the postoperative complications in day surgery group was lower than in inpatient surgery group (OR 0.20, 95% CI 0.16–0.24, *P* < 0.00001, I^2^ = 91%) (Fig. 6A). From the Fig. 6B–F, in DG, it showed that postoperative complications including hydrothorax, hemorrhage, arrhythmia, lung infection and persist air leak were less than in NG. However, there is no statistical difference between two groups in pneumothorax, chylothorax and hoarseness (Fig. 7A, C).

## Comment

Day surgery was first introduced by James Nicoll in 1909 [6]. The amount of day surgery in Europe and the United States has reached more than 80% of the total number of operations in their hospitals [11–13]. In the early days, the ERAS concept was more reflected in the optimization of the perioperative diagnosis and treatment process for patients undergoing general surgery [14]. Then, based on the application of laparoscopic minimally invasive technology, the positive role in the ERAS process is highlighted during the surgery [15]. Compared with traditional surgical methods, minimally invasive surgery itself has the advantages of less trauma, less pain, faster postoperative recovery and shorter hospital stay. It not only reduces the stress response and complications in patients after the surgery, but also significantly improves the satisfaction of patients [3]. Since 2006, the clinical application of the ERAS concept with minimally invasive methods in thoracic surgery has been gradually carried out, especially in the optimization of perioperative management paths for patients with diseases in thorax [15].

The successful implementation of the ERAS concept in thoracic surgery has made it possible to complete the surgery in the day surgery unit. At the same time, the thoracic day surgery is also a further concentrated embodiment of the implementation of the ERAS concept. 7 studies were included in our research and we conducted the first meta-analysis to explore and compare the clinical efficacy of thoracic day surgery and inpatient surgery in patient with lung cancer.

Firstly, the result of meta-analysis found that the age, smoking status of patient, pulmonary function tests and comorbidity before the day surgery were better than in inpatient surgery group. The main reason might be that patients in day surgery were younger and fewer smoking histories than the normal, further reflecting the objective fact that fewer patients had preoperative comorbidities with better lung function. Surgeons can use these findings to develop patient-specific treatment plans and select patients that are most likely to succeed with an accelerated discharge [4]. But when we reviewed the included references again, which specifically mentioned studies on these relative contents in Dong’s two articles, there is no statistical difference between day surgery group and inpatient group. This means that the basic conditions of patients in day surgery and inpatient group are basically the same, and it also shows that the patients of day surgery is comparable to inpatient group. Further analysis of the remaining references, due to the late implementation of day surgery in thoracic surgery for lung cancer, it can be found from their data (Patel et al. and Linden et al.) that the number of day lung cancer surgery patients accounted for a relatively small part of total patients, which may cause the imbalance of baseline data when comparing the two groups in their researches. As the understanding of day surgery for patients with lung cancer increases, we believe that more and more patients will be included in the day surgery process, rather than those who are simply highly selected.

Regarding the surgical methods and resection range of lobe in operation, our meta-analysis data showed minimally invasive surgical method was more popular in DG than in NG and segmentectomy in day surgery group was more than in normal surgery group. In recent years, minimally invasive thoracoscopic surgery (video-assisted thoracoscopic surgery, VATS) for the treatment of ground glass nodule has basically reached a consensus, which is also a foundation for the development of thoracic day surgery [5]. Traditional lobectomy has a greater loss of pulmonary function, while segmentectomy can better preserve more pulmonary function, which is more conducive to the recovery of postoperative pulmonary function and the improvement of patients' quality of life [12]. Especially, the segmentectomy mentioned in our data does not include the wedge resection. In addition, results showed that the operative time was shorter in day surgery group than in normal group. With the popularization of VATS, the proficiency of surgical operations has been continuously improved, which has significantly shortened the operation time.

It is important to achieve the most effective patient care and optimal patient outcomes by reducing the LOS and postoperative readmission rates for patients [11]. 30-day readmission after lung cancer surgery can place additional financial burdens on patients. Therefore, it is critical to assess patient discharge from a financial and clinical perspective and to reduce the risk of readmission and early death after lung cancer surgery. Shorter LOS is also associated with lower hospital costs [13]. In our study, there is no difference between two groups in 30-day readmission. However, the 30-day mortality was lower in DG than in NG according to the result of 3 studies. According the result of Dong’ studies in 2020 and 2021, the average hospital cost in DG was lower than in normal group. By optimizing the perioperative management process of patients with lung cancer surgery, the main purpose is to minimize medical intervention, increase medical services, and ensure patient safety. On the premise of integrating more humanistic factors into treatment, it is possible to accelerate patient recovery and improve patient satisfaction with medical care. In addition, postoperative pain control is also a factor affecting the length of hospital stay, and difficulty in perioperative pain control may also be a barrier to discharge on the first postoperative day. Methods to reduce pain during surgery, such as minimally invasive techniques and changes in intraoperative analgesia, such as thoracoscopic intercostal nerve blocks (TINBs) together with a postoperative combination of acetaminophen and NSAIDs, may facilitate early discharge [6].

The occurrence of postoperative complications is also an important indicator for evaluating short-term results after surgery. The complications after lung cancer resection are hydrothorax, hemorrhage, arrhythmia, lung infection, persist air leak, pneumothorax, chylothorax and hoarseness [1]. The results of our meta-analysis displayed that POD1 patients had fewer overall complications than the inpatient surgery group. In DG, it showed that postoperative complications including hydrothorax, hemorrhage, arrhythmia, lung infection and persist air leak were less than in NG. However, there is no statistical difference between two groups in pneumothorax, chylothorax and hoarseness. We analyzed the included references, which specifically mentioned studies on complications, including Dong’s two articles, Patel’s article, Linden’s article, and Geraci’s article. The percentage of day surgeries planned in these papers that were completed were 95%, 88.2%, 99.8%, 94.9% and 90.3% respectively. There were several reasons for this situation. Firstly, minimally invasive surgery for the lung cancer has obvious significance in reducing postoperative complications than open thoracic surgery. Secondly, these factors have driven the adoption of ERAS after thoracic surgery. The focus of the ERAS program is reducing the incidence of complications in patients and the postoperative hospital stay can be safely shortened.

There are several limitations in this meta-analysis. Firstly, the number of studies included and the simple scale were relatively small. All studies included for meta-analysis were retrospective observational studies and lacked high-quality randomized controlled trials. Secondly, the age, comorbidity before the operation, pulmonary function tests and 30-day mortality had significant heterogeneity. Potential factors that could explain that compared with inpatient patients, day surgery patients are selectively younger, and have better physical fitness, better preoperative indicators, with fewer postoperative complications.

### Publication of bias

**Fig. 8:**
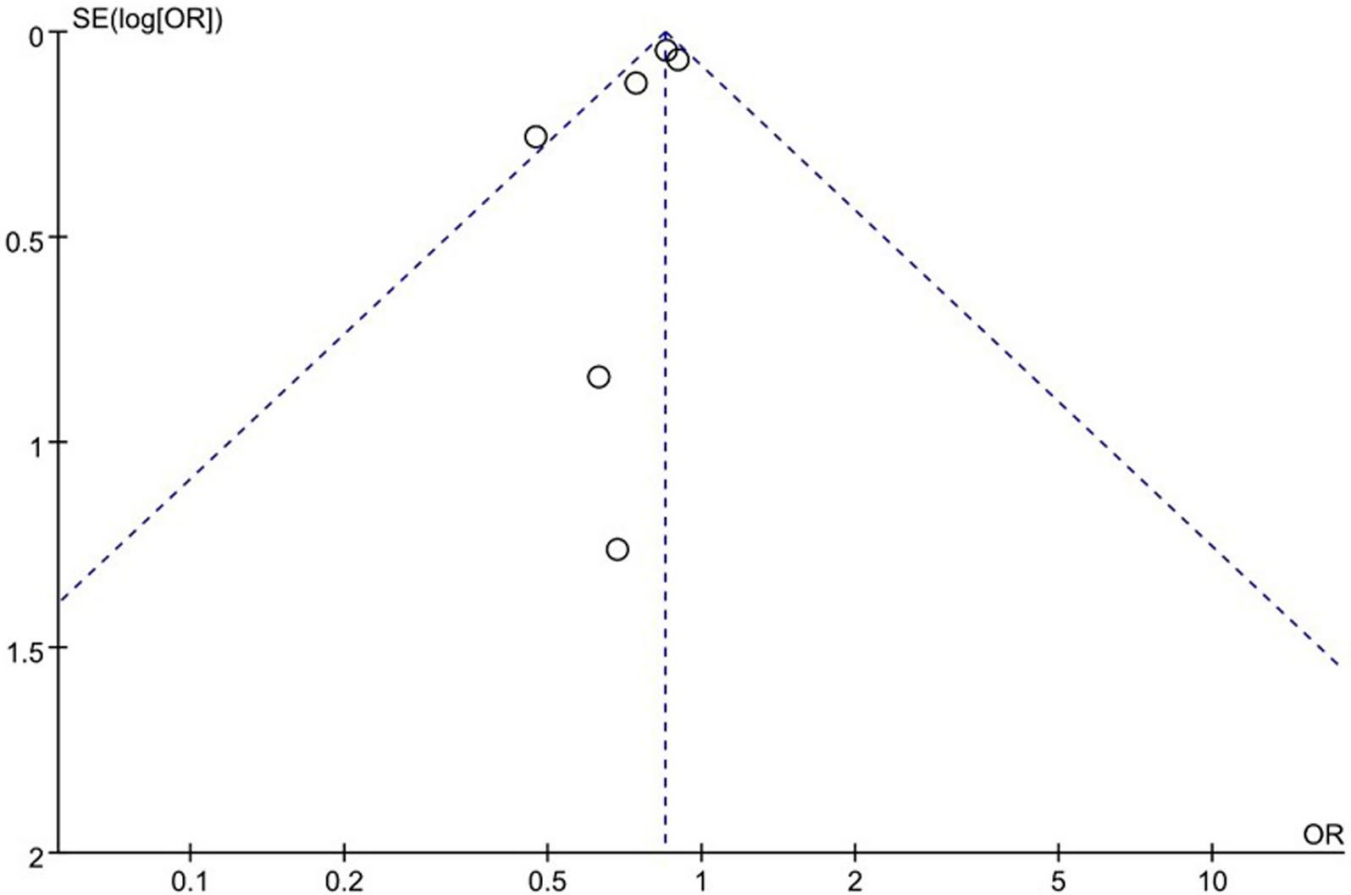
Funnel plot of the meta-analysis

A funnel plot of the overall complication was used to assess publication bias. The bilaterally symmetrical funnel plot of overall complication showed that no obvious evidence of publication bias was observed (Fig. 8).

## Conclusion

In summary, thoracic day surgery has more advantages over inpatient surgery in terms of length of postoperative hospital stay, operative time, average hospital cost, 30-day mortality and postoperative complications. We demonstrate that younger patients, patients receiving segmental resections by VATS, and those with better pulmonary function tests or without comorbidity can be discharged POD1 with low rates of complications and 30-day mortality, especial with ERAS program. More large-sample, high-quality studies are necessary to identify patient and institutional factors necessary for safe POD1 discharge in the future and day surgery for lung cancer incorporating the ERAS concept is a safe and effective modality.
